# Progressive Muscular Atrophy with Recovery

**Published:** 1888-09

**Authors:** J. Michell Clarke

**Affiliations:** Assistant-Physician to the Bristol General Hospital


### PROGRESSIVE MUSCULAR ATROPHY WITH RECOVERY.

By J. Michell Clarke, M.A., M.B., M.R.C.P., Assistant-Physician to the Bristol General Hospital.

The following case showed well-marked signs of the commencement of progressive muscular atrophy in the hands, and is especially interesting from the fact that complete recovery took place under anti-syphilitic treatment.

The patient was a leather-dresser of 30 years of age, of a healthy family; his father and mother were both alive. I first saw him on December 5th, 1887.

He had had syphilis some years previously, and an attack of rheumatic fever, not severe, in 1881. He had never suffered from any accident, and had always been temperate.

The present illness began two months previously with weakness and gradual loss of power in the thumb and index-finger of the right hand. At the same time he noticed that the muscles of the thumb and finger wasted. The weakness interfered with his work, and he was unable to write. There was at no time any loss of sensation, or any pain.

His face was pale and pasty, and he suffered from constipation. On examination, the thoracic and abdominal organs were normal. In the right hand the thenar eminence, the prominence corresponding to the abductor indicis, and the second and third dorsal interosseous spaces showed marked wasting; the eminences being flattened and the interosseous spaces hollowed out. There were also slight depressions between the flexor tendons in the palm of the hand. No fibrillation of the affected muscles was observed, but their mechanical irritability was increased, a tap causing a local contraction.

The extending action of the interossei in the fingers, as in making the up-stroke in writing, was entirely lost; while the action of the long flexors was perfect. The abducting action of the interossei was also lost, while adduction was extremely feeble.

The thumb could be opposed to the little finger, and could be flexed and extended; but all these movements were very weak.

The thumb-muscles gave diminished reactions to both the Faradic and Voltaic currents. In the abductor indicis the reaction to the Faradic current was almost entirely lost, a few fibres only responding to a very strong current. The grasp of this hand was exceedingly weak.

In the left hand there appeared to be slight wasting of the abductor indicis; and the special actions of the interossei were feeble.

There was nowhere any abnormal affection of sensation to be made out, either to pain, touch, or temperature.

The muscles of the shoulders, arms, and forearms were healthy; the tendon reactions normal. There was no affection of the legs, and the knee-jerks were normal.

The affected muscles were systematically rubbed night and morning, and a constant current of moderate strength applied for ten minutes every other day. During the time he was under treatment—four months—he was given a mixture containing arsenic and iodide of potassium, the dose of each being gradually increased.

At the end of seven weeks a decided improvement could be seen: the thenar eminence was much less flattened, and the interosseous spaces partly filled up again, while there was a marked gain of strength.

At the end of four months the strength of the hand and movements of the fingers were very good; the wasting had disappeared, except that the abductor indicis was a little smaller in the right hand than in the left; but the other muscles were of good size and tone.

The electrical reactions were normal.

He was now able to do his work well, and was anxious to return to it. His hand has remained well for six months, and is as strong as ever it was.

The lesion here would seem to have been a syphilitic one in the grey matter of the anterior cornu, affecting the ganglion-cells, and so situated as to exactly produce the symptoms of progressive muscular atrophy in those cases in which it begins in the small muscles of the hand. Complete recovery in cases of this kind, and recovery under anti-syphilitic treatment, is so rare that I have thought it worth while to record the case.

